# Health alliance for prudent prescribing and yield of antibiotics in a patient-centred perspective (HAPPY PATIENT): a before-and-after intervention and implementation study protocol

**DOI:** 10.1186/s12875-022-01710-1

**Published:** 2022-05-02

**Authors:** Anders Bjerrum, Ana García-Sangenís, Daniela Modena, Gloria Córdoba, Lars Bjerrum, Athina Chalkidou, Jesper Lykkegaard, Malene Plejdrup Hansen, Jens Søndergaard, Jørgen Nexøe, Ingrid Rebnord, Isabel Sebjørnsen, Jette Nygaard Jensen, Matilde Bøgelund Hansen, Katja Taxis, Maarten Lambert, Ria Benko, Beatriz González López-Valcárcel, Fabiana Raynal, Nieves Barragán, Pia Touboul, Pascale Bruno, Ruta Radzeviciene, Lina Jaruseviciene, Auste Bandzaite, Maciek Godycki-Cwirko, Anna Kowalczyk, Christos Lionis, Maria-Nefeli Karkana, Marilena Anastasaki, Jamie Coleman, Helena Glasová, Michiel van Agtmael, Pierre Tattevin, Alicia Borràs, Carl Llor

**Affiliations:** 1 Fundació Institut Universitari per a la Recerca a l’Atenció Primària de Salut Jordi Gol, Barcelona, Spain; 2 University of Copenhagen, Copenhagen, Denmark; 3 Department of Public Health, Research Unit for General Practice, University of Southern Denmark, Odense, Denmark; 4 NORCE Norwegian Research Centre AS, Bergen, Norway; 5 The Department of Clinical Microbiology, Copenhagen University Hospital – Herlev and Gentofte, Copenhagen, Denmark; 6 Rijksuniversiteit Groningen, Groningen, The Netherlands; 7 University of Las Palmas de Gran Canaria and Fundación Canaria Parque Científico Tecnológico, Las Palmas, Spain; 8 Spanish Society for Family and Community Medicine, Barcelona, Spain; 9 Department of Public Health, Nice University Hospital, Nice, France; 10 Ltd Mano Seimos Gydytojas (My Family Doctor), Klapeida, Lithuania; 11 Lithuanian University of Health Sciences, Kaunas, Lithuania; 12 Centre for Family and Community Medicine, the Faculty of Health Sciences, The Medical University of Lodz, Lodz, Poland; 13 Clinic of Social and Family Medicine, School of Medicine, University of Crete, Rethymno, Greece; 14 European Association for Clinical Pharmacology and Therapeutics, London, UK; 15 Rennes University Hospital, Rennes, France; 16 University Institute for Patient Care, Barcelona, Spain; 17 Institut Català de la Salut, Via Roma Health Centre, c. Manso, 19, 3rd floor, 08015 Barcelona, Spain

**Keywords:** Antimicrobial stewardship, Medical audit, Anti-bacterial agents, Primary health care, Nursing homes, Pharmacies, After-hours care

## Abstract

**Background:**

Excessive and inappropriate use of antibiotics is the most important driver of antimicrobial resistance. The aim of the HAPPY PATIENT project is to evaluate the adaptation of European Union (EU) recommendations on the prudent use of antimicrobials in human health by evaluating the impact of a multifaceted intervention targeting different categories of healthcare professionals (HCPs) on common community-acquired infectious diseases, especially respiratory and urinary tract infections.

**Methods/design:**

HAPPY PATIENT was initiated in January 2021 and is planned to end in December 2023. The partners of this project include 15 organizations from 9 countries. Diverse HCPs (doctors, nurses, pharmacists, and pharmacy technicians) will be audited by the Audit Project Odense (APO) method before and after an intervention in four different settings: general practice, out of hours services, nursing homes and community pharmacies in four high antibiotic prescribing countries (France, Poland, Greece, and Spain) and one low prescribing country (Lithuania). About 25 individuals from each professional group will be recruited in each country, who will register at least 25 patients with community-acquired infections during each audit period. Shortly before the second registration participants will undertake a multifaceted intervention and will receive the results from the first registration to allow the identification of possible quality problems. At these meetings participants will receive training courses on enhancement of communication skills, dissemination of clinical guidelines with recommendations for diagnosis and treatment, posters for the waiting rooms, and leaflets for patients. The results of the second registration will be compared with those obtained in the first audit.

**Discussion:**

HAPPY PATIENT is an EU-funded project aimed at contributing to the battle against antibiotic resistance through improvement of the quality of management of common community-acquired infections based on interventions by different types of HCPs. It is hypothesized that the use of multifaceted strategies combining active intervention will be effective in reducing inappropriate prescribing and dispensing of antibiotics.

**Study registration:**

EU Health programmes project database https://webgate.ec.europa.eu/chafea_pdb/health/projects/900024/summary; date of registration: 1 January 2021.

## Background

Excessive antibiotic use is considered to be the main driver of the growing development and spread of antimicrobial resistance (AMR) [1]. Infections caused by resistant bacteria are challenging as they can lead to increased mortality, prolonged hospital stays and higher medical costs [2, 3]. Countries with inappropriate and high use of antibiotics, such as the southern and eastern European countries, have a high rate of resistance [4]. As antibiotic resistance spreads across borders, high prevalence countries serve as a source of bacterial resistance for countries with a low prevalence [5]. Therefore, AMR is an important issue with a potentially serious impact on all countries.

The most effective measure to curb the problem of AMR is to reduce the unnecessary prescriptions of antibiotics is [6]. Most antibiotics in general practice (more than 80%) are prescribed for respiratory tract infections (RTI) and urinary tract infections (UTI) [7, 8]. Therefore, community-acquired infections (CAI) should constitute the main target of stewardship strategies. The development of detailed guidelines by the European Union specifically related to the prudent use of antimicrobials in humans (the EU AMR Guidelines) has ushered in a new phase of the overall strategy [9]. To intensively engage key stakeholders, it is necessary to increase knowledge and implement the EU AMR Guidelines and reduce the demand and prescription of antibiotics in inappropriate cases. In order to change both prescribing and dispensing habits and patient expectations stakeholders must be asked to re-evaluate well-established practices. The evidence shows that just delivering guidelines is not enough to restrict antibiotic prescribing [10]. Changing practice behaviour is challenging and requires the implementation of a systematic approach following components of the normalization process theory (NPT), in which individual and group reflection of the actions that need to be implemented to reduce inappropriate prescribing of antibiotics will secure the achievement of high impact and sustainable results [11].

Previous results of similar studies aimed at decreasing the inappropriate use of antibiotics have demonstrated that a reduction mainly depends on the context and the components of the multifaceted intervention. In the HAPPY AUDIT project, 440 general practitioners (GP) from six countries registered 47,011 consultations before and after an intervention including individual prescriber feedback, training courses, clinical guidelines, posters for waiting rooms, patient brochures and access to two different point of care tests [12]. After the intervention, clinicians significantly reduced the percentage of consultations resulting in antibiotic prescription, and in patients with lower RTIs, doctors reduced the prescribing rate by a percentage ranging from 42% in Lithuania to 9% in Argentina. In a quality control-based randomised clinical trial carried out in four South American countries, the clusters of practices assigned to the intervention groups reduced antibiotic prescribing rates from 37.4 to 28.1% in patients diagnosed with either acute otitis media or acute bronchitis, whereas the reduction in the control group was marginal, from 29.0 to 27.2% [13].

In the HAPPY PATIENT project, the interventions will be carried out in five countries with different cultural backgrounds and different organisations of healthcare: Greece, France, Spain, Poland, and Lithuania. This will ensure better generalisation of the results to a wide range of settings and different healthcare organisations. Table 1 describes the differences across these five countries [14]. In terms of antibiotic consumption, the first four countries have high levels of antibiotic consumption and according to the latest statistics on antibiotic prescribing in the primary care setting in 2020 these countries ranked in the first quartile, while Lithuania has a relatively low consumption level, that is under the European mean [7]. The project also analyses the dispensing process, as pharmacists play a crucial role in directly reinforcing messages about appropriate use when dispensing antibiotics to patients or carers and are amongst the groups most trusted to convey such messages.

**Table 1:** Characteristics of the health care systems in the HAPPY PATIENT project target countries

	France	Greece	Lithuania	Poland	Spain
Population (million)	66.9	10.7	2.8	38.4	46.7
Proportion of population 65+ (%)	19.8	21.9	19.7	17.2	19.3
Percentage of the gross domestic product to health	11.3	8.0	6.5	6.5	8.9
Number of doctors in practice per 1000 inhabitants	3.2	6.3	4.6	2.4	3.9
Number of nurses in practice per 1000 inhabitants	10.8	3.3	7.7	5.1	5.7
Predominant mode of provision	Private	Public	Public	Public	Public
Copayment of patients in primary care	Yes	No, public providers	No	No	No
Copayment of patients in prescriptions	Yes	Yes	Yes	Yes	Yes
Organisation in primary care	Groups of GPs and other HCPs or single-handed practices	Groups of GPs and other HCPs	Groups of GPs and other HCPs	Individual and group GPs and other HCPs	Groups of GPs and other HCPs
The patient must be registered with a GP	No, but they have advantages if registered	No	Yes	Yes	Yes
Freedom of choice of GP	Yes	Yes	Yes	Yes	Yes
Gatekeeping. GP referral requirement	For some specialist care	Not required	For most specialist care	For some specialist care	For most specialist care
Remuneration of GPs	Mix of salary, payment for service, and capitation	Mix of salary and payment for services	Mix of payment for services and capitation	Mix of capitation and payment for services	Mix of salary and capitation
GP mean income per year (in euros)	50,263	25,000	10,782	38,400	45,000
Average population size per GP	864	–	1550	1539	1500
Frequency of GP medical consultations per inhabitant and year	6.0	4.2	6.6	6.6	3.0
Status of long-term care providers	Mixed provider status: public (58%); not-for-profit organizations (religious groups, corporate org.) (26%); private (16%)	Mixed provider status: public sector (appr. 1/3); private, mainly for-profit and some not for-profit (ie. church charitable org. and local authorities) (appr. 2/3)	Mixed provider status: public (appr. 50%); private (appr. 50%)	Mixed provider status: public (appr. 70%); private sector (appr. 30%)	Mixed provider status: public (26.6%); private (71,9%)
Number of pharmacies per 100,000 inhabitants	33	88	40	36	47

*GP* General Practitioner, *HCP* Health Care Professional. Data from 2018

## Methods/design

### Aims of the study

The overall goal of the HAPPY PATIENT project is to increase the impact of EU recommendations on the prudent use and dispensing of antimicrobials in human health through a focus on critical interaction between patients and HCPs, evaluating the impact of a multifaceted intervention on their antibiotic prescribing or dispensing in four different settings.

### Design

**Fig. 1:**
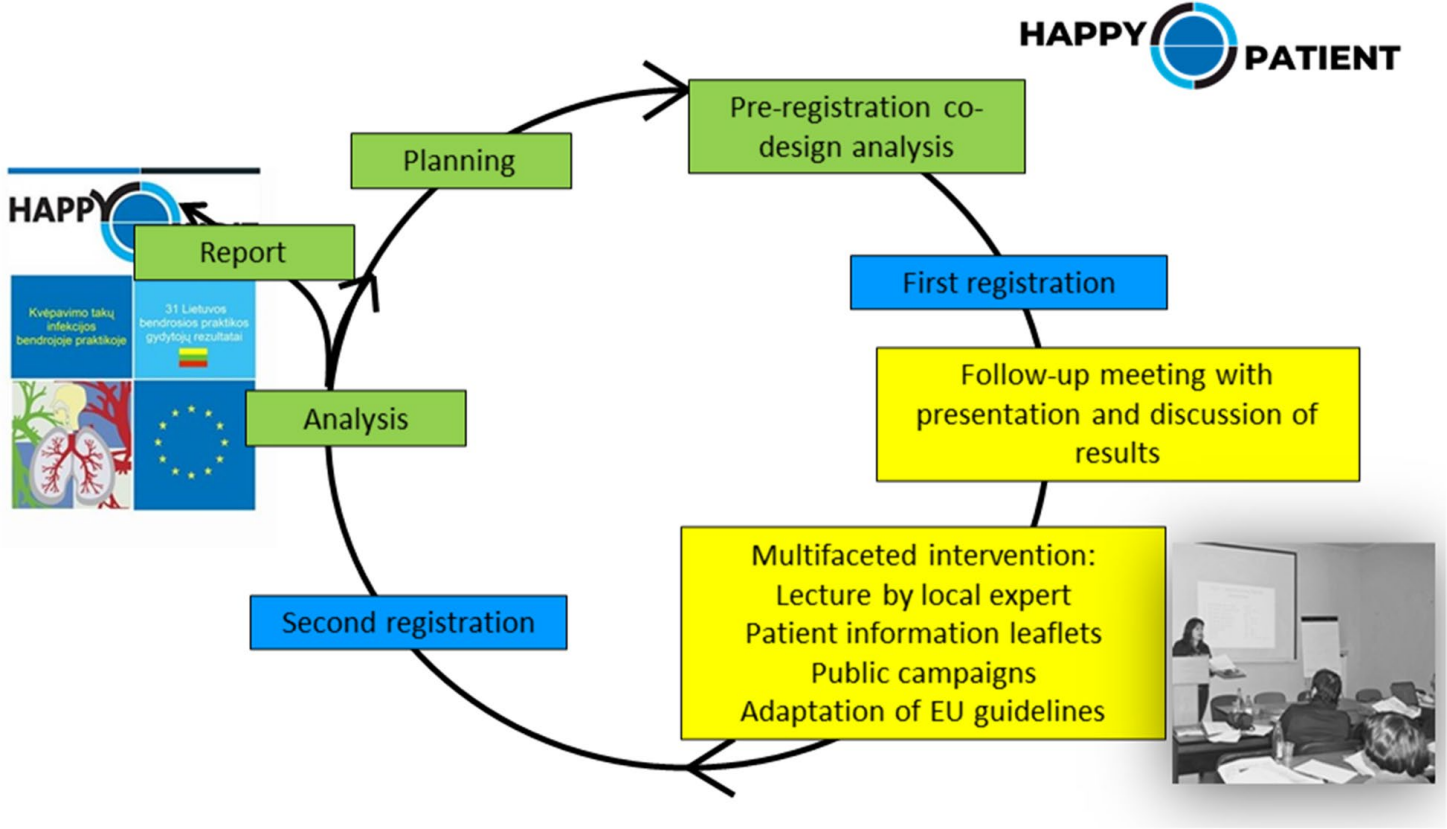
Audit Project Odense cycle in the HAPPY PATIENT project

HAPPY PATIENT was initiated in January 2021 and the project is planned to finish in December 2023. The project includes 15 partners from nine countries, as indicated in Table 2. The Audit Project Odense (APO) methodology is used [15], which is an evidence-based multifaceted intervention to improve quality of care by implementing guidelines [16]. This is a before and after intervention study, with two audit registration periods (Fig. 1). A minimum number of 25 HCPs will be recruited in each setting and will collect CAIs during a first audit registration in February 2022. In autumn 2022, the four groups of HCPs will be invited to a face-to-face meeting to receive a multifaceted intervention. In February 2023, the same participants will register the same type of CAIs for a second audit.

**Table 2:** Partners in HAPPY PATIENT

Participant organisation name	Abbreviation	Role
Institut Català de la Salut and Fundació Institut Universitari per a la Recerca a l’Atenció Primària de Salut Jordi Gol	ICS/IDIAP	Coordinator of the project. Spanish network
University of Copenhagen	UCPH	Co-design and consensus methodology
Research Unit for General Practice Odense	RUPO	APO methodology. Primary healthcare settings
NORCE Norwegian Research Centre AS	NORCE	Out of hours settings
The Capital Region of Denmark	CAPREG	Nursing homes settings
Rijksuniversiteit Groningen, The Netherlands	RUG	Pharmacy settings
University of Las Palmas de Gran Canaria and Fundación Canaria Parque Científico Tecnológico	ULPGC/FCPCT	Analysis and evaluation of the results
Spanish Society for Family and Community Medicine	SEMFYC	Dissemination and training in communication skills
Nice University Hospital	CHUNICE	French network
Ltd Mano Seimos Gydytojas (My Family Doctor)	FDC	Lithuanian network
Medical University of Lodz, Poland	MUL	Poland network
University of Crete, Greece	UOC	Greek network
European Association for Clinical Pharmacology and Therapeutics	EACPT	Advisor
Rennes University Hospital, France	CHURE	Advisor
University Institute for Patient Care, Spain	UIC	Advisor

### Setting and population

We will carry out the audit registrations and monitor the intervention outcomes in five countries (Greece, France, Spain, Poland, and Lithuania) with diverse antibiotic prescription rates. In each country, data will be collected in four different settings: (a) general practice, including GPs and nurses; (b) secondary care, in out-of-hours (OOH) services, including doctors and nurses; (c) tertiary care, with nursing homes, involving doctors and nurses who directly engage patients in relation to the prescribing and use of antibiotics; and (d) community pharmacies, with pharmacists and pharmacy technicians.

### Project phases

- Phase 1 - Codesign study: June–November 2021
- Phase 2 - Pilot test of the audit registration charts: November 2021
- Phase 3 - First audit registration: February 2022
- Phase 4 - Multifaceted intervention: October–December 2022
- Phase 5 - Second audit registration: February 2023
- Phase 6 - Results: November 2023

### Phase 1: co-design process

The project co-design process, using a modified Delphi technique, was used to incorporate the opinion of different HCPs in the development of the educational material that will help facilitate the communication between HCPs and patients when discussing the need and use of antibiotics for common CAIs [16, 17]. HCP “experts” from the four different sectors in the HAPPY PATIENT project across the five target countries were contacted by local coordinators and constitute the panel of “experts” that will prioritize areas of improvement.

The expert panel process includes the following steps: (a) review of scientific literature and publicly available surveys at European and national level regarding knowledge gaps associated with inappropriate use of antibiotics in CAIs; (b) first draft of a list of themes based on a literature review; (c) first prioritization round, during which the identified panel of “experts” receive a list of statements and knowledge gaps covering the following domains: knowledge gaps and misconceptions about antimicrobial resistance, use of antibiotics in general, and use of antibiotics for CAIs, namely RTIs and UTIs, and rate each statement on a seven-point Likert scale; (d) individualized feedback; the experts are given individualized feedback in the form of bar charts showing the distribution of ratings from the first round, with the experts’ rating highlighted in the figure and distributed by sector, so differences across countries within the same sector can be discussed; (e) consensus meetings – the panel of experts participate in a discussion about which of the knowledge gaps and misconceptions included in the Delphi study lead to increased inappropriate use of antibiotics in each sector; (f) second assessment of themes, with those reaching > 80% consensus are summarized in the different types of communication and intervention material; (g) iterative modification, during which the panel of experts and identified patient associations receive the first draft of the communication material and participate in a process of discussion, rephrasing and iterative evaluation of the material, resulting in the final version of the communication material. Lastly, on the basis of the NPT framework that uses collective participation and reflection to ensure the success of the intervention and to lead to change in the dimension of changing practice behaviour of the HCPs, the result of the co-design process will be used for the training, audit and workshops that will be conducted during the multifaceted intervention and the APO cycles [17].

### Phase 2: audit registrations

The project will conduct two audit registrations: the first will begin before the intervention (February 2022) and the second will take place after the intervention (February 2023). The same month has been chosen for the implementation of both registrations to minimize the effect of potential seasonal variation in CAIs. A registration pilot testing, in November 2021 with approximately five HCP participants per setting and per country, ensured that the content of the registration chart was relevant to their practice and easily understood, and confirmed that sufficient patients could be recruited.

**Fig. 2:**
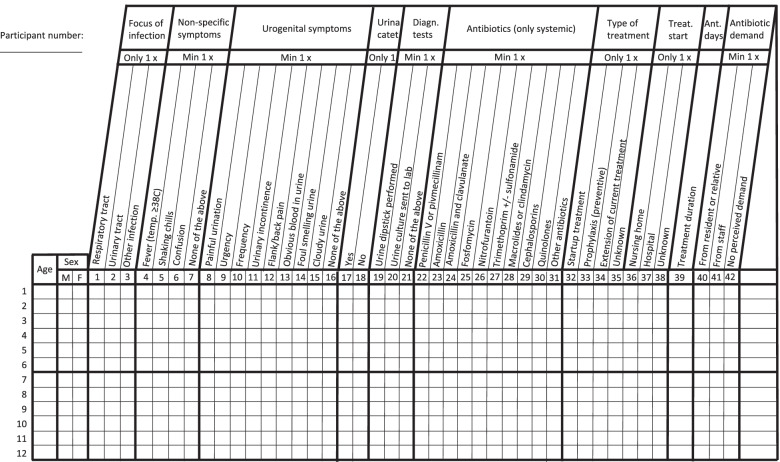
Draft of the template to be used in nursing homes in the HAPPY PATIENT project (patients receiving antibiotics).

During the audit period the participants will use the APO registration chart to record all consultations or dispensations regarding the CAIs selected for audit. Participants will be asked to fill in the chart with the consecutive consultations they receive regarding the CAIs selected, and in the case of pharmacies, all the systemic antibiotic dispensations given for acute infections. A specific registration chart for each of the 4 settings has been developed considering their characteristics and the quality improvement areas that HAPPY PATIENT seeks in each setting. The example of the template used in nursing homes is depicted in Fig. 2. At most, 10 main domains and a maximum of 45 variables are used to describe the topic investigated. The main domains are lined up in a logical way; for example: type of contact, symptoms present, examinations performed, diagnosis, treatment, and assessment. According to the APO methodology the variables are exhaustive (include all possibilities) and exclusive (no overlapping), and all follow the same logic plan. One line is filled in for each registered case and, as a general rule, only multiple choice is allowed (no open-ended text). At least one choice per main group is needed. A short instruction sheet will be provided for all HCP participants, with details about the registration period, the inclusion and exclusion criteria for the cases and a brief explanation of the content of each main group.

**Fig. 3:**
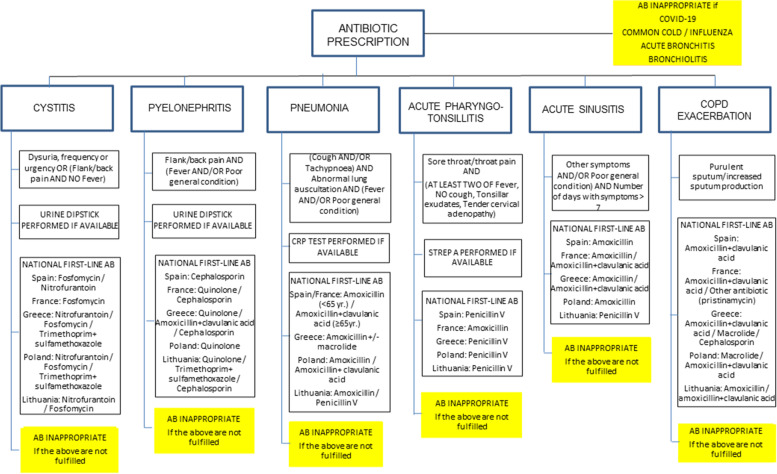
Defining antibiotic appropriateness in out of hours services in the HAPPY PATIENT project

Data originating from the registration will be used for quality assessment using quality indicators. In the case of general practice, OOH services and nursing homes, an indicator of antibiotic inappropriateness is considered. Antibiotic prescribing is considered appropriate if it fulfils the following criteria: a) the diagnosis for which this is given might be bacterial; b) it is suspected that the criteria presented by the patient are caused by bacteria; and c) the choice of the antibiotic reflects the local pathogens and resistance patterns in each setting. Anything else is inappropriate. Although antibiotic underprescribing – withholding antibiotic use when the infection is suspected to be of bacterial origin – is also inappropriate, this is negligible in western countries according to the medical literature [18]. Based on the charts used in the registrations the group defined when an antibiotic was appropriate or not. In the case of pharmacies, several indicators have been defined, based on the safety and the appropriate advice given to the patients. Figure 3 describes the example used in OOH services.

### Phase 3: multifaceted intervention

In each of the five target countries, face-to-face or online training workshops plus a few online courses will be given to each of the four groups of HCPs. These will take place approximately 3 months before the second registration (October–December 2022) and will be run by the local coordinators of the project. In these workshops, the HCPs will discuss the results of the first audit and set goals for antibiotic prescribing or dispensing based on the audit results, expert involvement, and the EU AMR Guidelines. The elements of this multifaceted intervention are described in the Table 3.

**Table 3:** Components of the multifaceted intervention

• Provision of individual prescriber or dispenser feedback of the results of the first registration at an individual and group level, identifying potential quality problems from the first registration reflecting the quality of their own care provided and giving peer-to-peer feedback.	
• Training course on the appropriate use of antibiotics for common CAIs, with clinical guidelines on recommendations for the diagnosis and treatment of infections.	
• Enhancement of communication skills to be used during the consultation or dispensation with patients with CAIs.	
• Training material on evidence-based management of CAIs with an explanation of the natural course, management, and safety netting.	
• Posters for waiting rooms focused on the appropriate use of antibiotics.	
• Educational material on the rational use of antibiotics for patients, such as brochures and handouts about prudent use of antibiotics and an explanation of the concept of the antibiotic footprint. This material will be accessible at the point of contact and during the encounter with the HCP.	

The proposed multifaceted intervention seeks to address all the dimensions of the NPT by facilitating open discussion on the variation in practice behaviour and strengthening the communication between the HCPs and the patients. Strengthening communication is a key element of our patient-centred approach. Several studies have shown that most patients do not necessarily want an antibiotic prescription but want time to talk to the HCP about their concerns [19]. Communication skills training includes strategies to address patient concerns regarding diagnosis, prognosis, benefits, harms of antibiotic treatment, how to manage patient expectations for antibiotics and provide alternatives during a clinical encounter. Therefore, communication training, providing HCPs with succinct and understandable arguments to communicate with their patients, and explanation guidelines is anticipated to decrease antibiotic use [20]. Reports comparing the results of the two audits and showing the progress on meeting the quality indicator goals will be produced, while participants will meet for a second round of peer-to-peer feedback and further goal setting.

### Limitations of the study

The use of the APO method has some limitations. Perhaps the most important limitation of the data collected is the lack of external validity. HCPs participate on a voluntary basis and their prescribing or dispensing habits might not represent the average use of antibiotics in their country. Additionally, HCP providers participating in audits may be more interested in quality development and might have a more rational use of antibiotics than non-participating HCPs [21]. Another limitation which should be considered is the fact that performing an audit may itself influence providers’ prescribing or dispensing behaviours when HCPs know they are being observed, leading to the, so-called, Hawthorne effect.

The amount of time needed to be involved in a quality improvement project could be a relevant barrier to participation. However, while filling out one registration takes less than 2 min, HCPs need to set aside time for the educational courses or other activities planned in the intervention. The APO methodology has been widely used in general practice, but not as much in OOH services and nursing homes as to our knowledge, this is the first time it will be used in community pharmacies. The cross-sectional nature of the APO method is another weakness. Variables included in the registration chart are lined up in a certain way to align with the consultation or dispensation process. Theoretically, the decision to treat should be taken after a diagnosis has been established. In general practice, however, the diagnostic procedures and the decision to treat are intertwined [22]. HCPs may decide whether to prescribe a drug at the same time, or even before. After making the decision to prescribe, clinicians may thus adjust the diagnosis to fit the decision about treatment. This may lead to a diagnostic misclassification bias. However, this potential bias will affect the validity of the diagnosis both before and after the intervention and the likelihood of influencing the effect of the intervention is small.

Due to the limited time allocated for the registration process, only the typical signs and symptoms for RTIs and UTIs will be collected. This may lead to some limitations. Non-biomedical factors that may represent powerful predictors of antibiotic prescription are not considered in this study. This limits the definition used for antibiotic appropriateness in our study as this is based on the different items collected in the registration charts. Another limitation is the lack of studies using the APO method in the pharmacy setting. In addition, the use of over-the-counter antibiotics, still available in some European countries, is not covered in this study.

### Statistical analysis plan

Statistical methods for inference in small samples will be used according to a statistical analysis plan. The minimum number of HCPs per APO audit cycle has been estimated based on the assumption that each HCP will register about 25 cases, including either consultations or dispensations, during the audit registration. A decrease of 15% in antibiotic prescribing can be expected after the intervention (from about 40% before to about 25% after). The within-practice correlation coefficient is 0.1. If the comparison is to be performed at the 5% level of significance (two-sided) with 80% power, then the number of HCPs to be included should be 25 per APO cycle. There will be 20 APO cycles in the project (one for each of the 4 healthcare settings involved in each of the 5 target countries). We assume the participation of 500 HCPs in the project and the registration of 25,000 cases.

We will apply the Fisher exact test to the frequencies of prescriptions before and dispensations before and after the audit for each group of professionals and setting/country to test the null hypothesis of no effect of the audit. We will also estimate the effect of the interventions with logistic two-level regression models: a) cases of CAIs and b) HCP participants. Antibiotic prescription will be considered as the dependent variable (yes/no) as well as the concepts of inappropriate prescribing and inappropriate dispensing. These models control for symptoms and duration, as well as for the demand for antibiotics by the patient. Goodness of fit will be assessed using the Wald test of the model, with the deviance test to compare alternative models. Statistical significance will be considered with *P* < 0.05. The data will be analysed using Stata v16.

### Data management

All audit data will be collected on paper. The registration templates will be stored securely by each HCP taking part, being only accessible by trial staff and authorised personnel. Once the audit is finished, the registration forms will be transferred to the University of Southern Denmark and will be entered into a database. Data from patients will be anonymously collected from the beginning and no identifiable or personal information about patients will be gathered. HCP participants will be identified with a number. Identifiable data of the HCP (e-mails and the identifier number) will be kept confidential in a separate file and will only be accessible to authorised personnel who require the data to fulfil their duties within the scope of the research project, complying with General Data Protection Regulation requirements.

Data collection will be performed once the HCP has signed the informed consent form. We will only retain participant e-mails and the basic information about the HCP participants during the time this study is running. Anonymised data from the survey will be stored for a minimum of 5 years after publication of the results. Anonymised project data will be shared for common analyses and presentation to the scientific community through publications and conferences. The institutions responsible for the treatment of the data are the coordinator and the institution leading the APO methodology.

### Ethics and dissemination

The study will be conducted in accordance with the protocol, the Declaration of Helsinki, the principles of Good Clinical Practice, The General Data Protection Regulation (EU) 2016/679 and the Human Research Act as well as other locally relevant regulations. This project has an Ethical Advisor that prepared the necessary documents to submit the project to the Ethics Committee Boards of the different participating countries. The Ethical Committee Boards of Greece and Spain deemed necessary to review the project and gave their favourable opinion. In Lithuania, Poland and France there was no need for the Ethical Committee to review this type of projects. This was confirmed by the national coordinators together with their Ethical Committee Boards.

HCPs will be contacted by local coordinators who will explain the study and hand over the information sheet to the professional, giving the professional time to read it and ask any questions. If HCPs accept to take part in the study, they will sign the consent form before participation. Participants will be able to withdraw from the project at any time without giving any explanation.

Information gathered from the patient consultations or dispensations will be personal non identifiable data (only age in years and sex will be recorded) and the minimum data set necessary for the study will be collected. No intervention or follow-up will be performed in the patients except for the distribution and discussion of leaflets on infections. Data will be anonymised from the beginning, coded in the template with the code of the HCP and a consecutive number for each consultation or dispensation. There will be no intervention, no follow-up and no documents that link the patient information with its code. Therefore, individual patient consent is not required. The HAPPY PATIENT project is an intervention study targeting HCPs to improve their performance according to good clinical practice and it does not involve any risk to the HCPs nor the patients whose CAI consultation or dispensation data are collected. Therefore, insurance is not needed for the HAPPY PATIENT project.

The final findings of this quality improvement project will be submitted to a peer-reviewed journal and will be presented in international scientific conferences. The results will be used to inform public health interventions on appropriate antibiotic prescribing and dispensing. Data from all centres will be analysed together and published as soon as possible. The HCPs participating in the study will receive a summary of the main project findings and the perspectives. All data collected as part of this quality improvement project will be shared for common analyses and presentation to the scientific community through publications and conferences.

## Discussion

AMR is a growing problem that threatens social development, human and global health. Infections caused by antibiotic-resistant bacteria are associated with a greater need for medical visits, more hospital admissions, higher mortality, and higher economic costs. Reducing the unnecessary prescription of antibiotics has been shown to be the most effective measure to curb the problem of AMR. Despite the high use of antibiotics and the growing development of AMR, only a few initiatives have been carried out to reduce the inappropriate use of antibiotics in more than one setting. The aim of the HAPPY PATIENT study is to evaluate the impact of a multifaceted intervention programme focusing on appropriate treatment of CAIs and targeting different types of HCP who constitute the first contact with the patients (doctors, nurses, pharmacists) and patients in the community. To our knowledge no other study has taken all these sectors into account. In addition, the study takes place in five European countries with diverse prevalence of antibiotic resistance, and with different cultural backgrounds and different healthcare organisations, increasing the extrapolation of the findings to a wide range of settings. The intervention will combine several techniques, including feedback to HCPs, training courses, follow-up meetings, discussion of guidelines, enhancement of communication skills, posters for waiting rooms and leaflets to the patients. We anticipate that inappropriate antibiotic prescribing and dispensing will be lowered after the intervention in the four settings, as has been shown in other studies with the use of the same APO methodology [12, 13].

The APO cycles in this study will be performed in a real-life practice setting and patients will not be informed about the project prior to the consultations or dispensations. HCPs participating in the audit will not be allocated extra time for consultations or dispensations, and they will not be able to make considerable changes in their practice activities during the registration. Thus, they will see the patients in the same way they would if not participating in the audit.

## Data Availability

No data sets exist nowadays. All open results from HAPPY PATIENT are expected to be stored in an Open Access repository, the Zenodo platform (http://zenodo.org). Zenodo is a EU-backed portal, linked to the OpenAIRE initiative (https://www.openaire.eu/, with a Digital Object Identifier (DOI) system (http://www.doi.org). All open data will also be available at the webpage of the project (https://happypatient.eu/).

## Abbreviations

AMR: Antimicrobial Resistance
APO: Audit Project Odense
EU: European Union
GP: General Practitioner
OOH: Out of Hours
HCP: Health Care Professional
CAI: Community-Acquired Infection
NPT: Normalisation Process Theory
RTI: respiratory tract infection
UTI: urinary tract infection
